# Case Report: Granular cell tumor of the axillary tail mimicking node-positive breast carcinoma — two cases with imaging–pathology correlation

**DOI:** 10.3389/fonc.2026.1888898

**Published:** 2026-06-11

**Authors:** Yuming Shao, Yang Xu, Shasha Song, Hao Lin, Jie Zhang

**Affiliations:** 1 Department of Breast and Thyroid Surgery, Shandong Provincial Maternal and Child Health Care Hospital Affiliated to Qingdao University, Jinan, China; 2 Department of General Surgery, Rushan People’s Hospital, Rushan, China; 3 Department of Pathology, Shandong Provincial Maternal and Child Health Care Hospital Affiliated to Qingdao University, Jinan, China; 4 Department of Critical Care Medicine, Shandong Provincial Maternal and Child Health Care Hospital Affiliated to Qingdao University, Jinan, China

**Keywords:** axillary tail, breast neoplasm, case report, core needle biopsy, granular cell tumor, imaging–pathology correlation, immunohistochemistry, perineural tumor growth

## Abstract

Granular cell tumor of the breast (GCTB) is a rare Schwann-cell-derived neoplasm whose multimodal imaging features overlap extensively with those of invasive carcinoma, frequently exposing patients to unnecessarily extensive surgery in the absence of preoperative tissue diagnosis. We report two institutional cases with detailed imaging–pathology correlation. In Case 1, a 53-year-old postmenopausal woman with a 2.5-cm left axillary tail mass displayed convergent malignant-pattern features across all three imaging modalities — a BI-RADS 5 spiculated mass with skin retraction on mammography, marked posterior acoustic shadowing on ultrasound, and heterogeneous enhancement with a type II (plateau) kinetic curve on contrast-enhanced MRI (a pattern associated with intermediate-to-high suspicion for malignancy) — together with an ipsilateral axillary lymph node demonstrating type III (washout) kinetics. The composite preoperative picture was clinically indistinguishable from cT2N1M0 invasive carcinoma. After the patient declined preoperative core needle biopsy (CNB), breast-conserving surgery and sentinel lymph node biopsy were performed; final pathology confirmed benign GCTB (S-100+, SOX10+, CD68+, Pan-CK−, GATA3−, ER/PR/AR−; 0 of 6 Fanburg-Smith criteria) with reactive nodal hyperplasia. In Case 2, a 38-year-old premenopausal woman with a 0.9-cm right breast nodule underwent ultrasound-guided vacuum-assisted biopsy (VAB) yielding benign GCTB; histology demonstrated tumor cells encircling peripheral nerve bundles within the lesion — direct morphological evidence supporting the established Schwann-cell origin of the tumor. Both patients remained disease-free at 12 and 36 months. These cases reinforce that preoperative CNB with a targeted immunohistochemistry panel — explicitly distinguishing GCTB from ectopic axillary breast carcinoma — should be strongly recommended for any suspicious axillary tail mass; Case 1, in which the patient declined CNB and proceeded directly to surgery, illustrates by counter-example the diagnostic uncertainty that results when this step is omitted. We present these cases in accordance with the CARE reporting guidelines.

## 1 Introduction

Granular cell tumor (GCT) was first described in 1926 by Abrikossoff, who originally postulated a myogenic origin and coined the term “granular cell myoblastoma” (1). Subsequent immunohistochemical and ultrastructural studies established the neuroectodermal derivation of GCT, with consistent expression of S-100 protein, SOX10, CD68, and neuron-specific enolase supporting an origin from Schwann cells of peripheral nerves (2, 3). More recently, Pareja and colleagues demonstrated recurrent inactivating somatic mutations in the endosomal pH regulators ATP6AP1 and ATP6AP2 in approximately 72% of GCTs, providing molecular underpinning for both the histogenesis and the cytoplasmic granular phenotype of the tumor (4).

Approximately 5–8% of all GCTs arise in the breast, where granular cell tumor of the breast (GCTB) accounts for only 0.03–0.15% of primary breast neoplasms (2, 5). Although most GCTBs are clinically benign, approximately 1–2% undergo malignant transformation, and synchronous breast carcinoma is reported in 6–10% of patients, mandating definitive preoperative histological characterization in every case (3, 6). The dominant clinical hazard of GCTB lies in its extensive radiological mimicry of invasive carcinoma. Across published series, the majority of GCTBs are reported as BI-RADS 4 or 5 lesions with spiculated margins, posterior acoustic shadowing, and heterogeneous enhancement on contrast-enhanced MRI with kinetic curves that overlap with malignancy (7, 8). In the single-institution series of Abreu et al., all five pathologically confirmed GCTBs presented as BI-RADS 5, with one demonstrating a washout curve on MRI (8). This high pre-test probability of malignancy on imaging frequently translates into unnecessarily extensive surgery when histological confirmation is omitted.

The diagnostic challenge is amplified when GCTB arises in the axillary tail of Spence, where a primary lesion with adjacent reactive lymphadenopathy may produce a composite imaging picture indistinguishable from locally advanced node-positive breast carcinoma. Carcinoma of the axillary tail of Spence and invasive carcinoma in ectopic axillary breast tissue must also be considered in this anatomical setting, given their fundamentally different oncological implications (9, 10). From the histogenetic standpoint, although the Schwann-cell origin of GCT is widely accepted on immunohistochemical and molecular grounds, direct morphological demonstration of tumor cells in intimate apposition to peripheral nerves remains valuable corroborating evidence, particularly in the breast, where such observations are infrequently documented (3, 11).

We report two institutional cases of GCTB that jointly illustrate two clinically consequential dimensions of the entity: (i) preoperative multimodal imaging features in the axillary tail that are radiologically indistinguishable from invasive node-positive carcinoma, and (ii) histologically demonstrated peripheral nerve encasement by tumor cells, providing direct morphological support for the Schwann-cell origin of GCTB. Both cases are presented with detailed imaging–pathology correlation and in accordance with the CARE (CAse REport) guidelines.

## 2 Case description

### 2.1 Case 1: axillary tail GCTB mimicking cT2N1M0 invasive breast carcinoma

A 53-year-old postmenopausal woman with no personal or family history of breast or ovarian malignancy presented in October 2024 with a 5-day history of a self-palpated painless mass in the left breast. Menarche was at age 13, menopause at age 51, and she had two full-term deliveries with no exogenous hormone exposure. Physical examination revealed a firm, fixed, 2.0 × 2.0 cm mass at the 2 o’clock position of the left axillary tail with overlying skin dimpling. No palpable axillary lymphadenopathy was noted. Complete blood count, hepatic and renal function, and serum tumor markers (CA15-3, CEA) were within normal limits.

**Figure 1:**
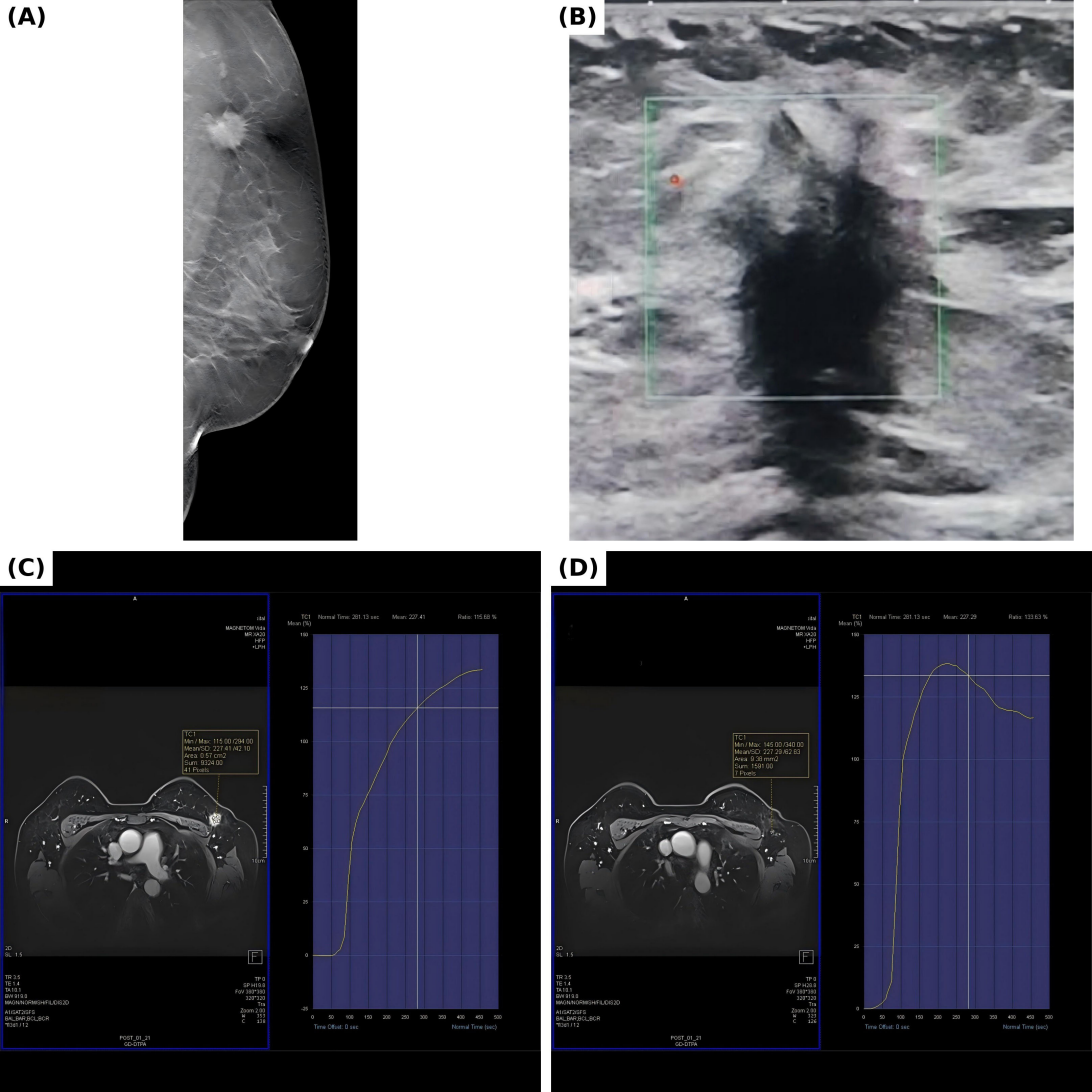
Multimodal imaging findings of case 1 — an axillary tail granular cell tumor of the breast clinically and radiologically indistinguishable from cT2N1M0 invasive carcinoma. **(A)** Mammography (mediolateral-oblique view): high-density spiculated mass in the upper portion of the left axillary tail with overlying skin retraction (BI-RADS 5). **(B)** Breast ultrasound: irregular hypoechoic mass measuring 1.8 × 1.7 × 1.4 cm with marked posterior acoustic shadowing (BI-RADS 4c); the lesion is bounded by the green region-of-interest (ROI) box generated by the ultrasound elastography acquisition. The disproportionate degree of acoustic shadowing relative to lesion size reflects the dense fibrous stroma of GCTB. **(C)** Contrast-enhanced axial breast MRI (T1-weighted post-gadolinium, fat-suppressed) of the primary lesion: an irregular enhancing mass in the left axillary tail (right side of the image; ROI marker shown adjacent to the lesion in the kinetic display) with marked heterogeneous enhancement. The right-hand panel displays the corresponding time–intensity curve generated from the ROI placed in the most rapidly enhancing area of the lesion; the curve shows rapid initial enhancement (initial slope > 100% increase) followed by a stable signal-intensity plateau (type II/plateau kinetics) — a pattern associated with intermediate-to-high suspicion for malignancy on ACR BI-RADS MRI assessment. **(D)** Contrast-enhanced axial MRI at the level of the ipsilateral left axillary lymph node (0.6 × 0.4 cm; ROI placed within the small enhancing node); the corresponding time–intensity curve demonstrates rapid initial enhancement followed by a clear decline in signal intensity in the delayed phase (type III/washout kinetics) — formally fulfilling radiological criteria for metastatic involvement. Sentinel lymph node biopsy subsequently demonstrated only reactive hyperplasia, illustrating the diagnostic trap of reactive lymphadenopathy associated with axillary tail GCTB.

Mammography demonstrated a high-density spiculated mass in the left axillary tail with skin retraction, classified as BI-RADS 5 (**Figure 1A**). No suspicious microcalcifications were identified. Breast ultrasound demonstrated an irregular hypoechoic mass measuring 1.8 × 1.7 × 1.4 cm with marked posterior acoustic shadowing, classified as BI-RADS 4c (**Figure 1B**). Contrast-enhanced breast MRI revealed an irregular mass with marked heterogeneous enhancement and a type II (“plateau”) time–intensity curve (TIC) using the ACR BI-RADS MRI kinetic classification (**Figure 1C**). Critically, MRI also identified an ipsilateral left axillary lymph node measuring 0.6 × 0.4 cm with a type III (“washout”) TIC pattern (**Figure 1D**). The composite preoperative picture — a BI-RADS 5 spiculated primary lesion with skin retraction and an axillary node with washout kinetics — was clinically indistinguishable from cT2N1M0 invasive breast carcinoma.

The case was discussed at a formal institutional multidisciplinary team (MDT) meeting on the day of the imaging review, with the conclusion entered into the structured MDT module of the hospital electronic medical record. Although the imaging composite was strongly suggestive of invasive carcinoma, the MDT explicitly flagged GCTB and invasive carcinoma arising in ectopic axillary breast tissue as alternative diagnoses — the rationale being that, although uncommon, GCTB is a recognised radiological mimic of carcinoma whose spiculated, shadowing, infiltrative imaging pattern is essentially indistinguishable from malignancy, and that the management implications of a benign Schwann-cell tumour, of carcinoma of the axillary tail of Spence, and of carcinoma in ectopic axillary breast tissue diverge so fundamentally that none could be excluded on imaging alone, and recommended preoperative core needle biopsy (CNB) with a targeted immunohistochemistry (IHC) panel before definitive surgical planning. The patient and her family declined CNB owing to anxiety regarding procedural pain and a perceived theoretical risk of tumor seeding. The surgical team re-counselled the patient on two separate occasions (in the clinic and on the day prior to surgery), explicitly emphasising the diagnostic value of CNB, the very different management implications of GCTB versus invasive carcinoma, and the negligible real-world seeding risk reported in the breast literature. A written informed refusal of CNB, co-signed by the patient and the surgeon, was archived in the medical record per institutional shared decision-making policy.

**Figure 2:**
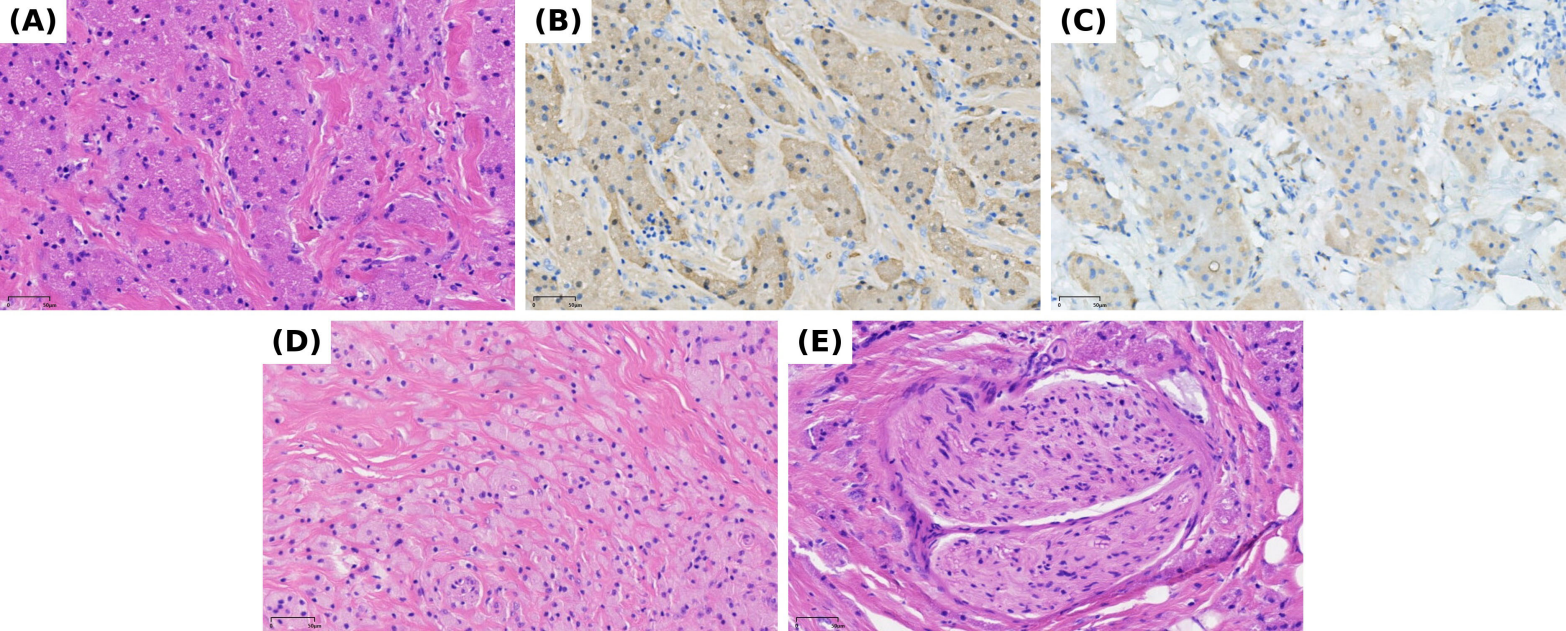
Histopathology and immunohistochemistry of both cases (scale bar = 50 µm in each panel). Case 1: **(A)** Hematoxylin and eosin stain (×200): tumor cells arranged in nests and cords with abundant eosinophilic granular cytoplasm and small, regular nuclei; the infiltrative tumor border extending into adjacent stroma contributes to the spiculated radiological appearance and mimicry of invasive carcinoma. **(B)** S-100 protein immunohistochemistry (×200): diffuse strong cytoplasmic and nuclear positivity — the primary immunohistochemical discriminator between GCTB and breast carcinoma, the latter being consistently S-100 negative. **(C)** CD68 immunohistochemistry (×200): diffuse cytoplasmic positivity, consistent with the lysosomal granular content of GCT and supportive of the Schwann-cell lineage. SOX10 immunohistochemistry was also strongly positive in Case 1, but a publication-ready image was not retrievable from the digital pathology archive (see Limitations, Section 3.5). Case 2: **(D)** Hematoxylin and eosin stain (×200): round to polygonal tumor cells with abundant eosinophilic granular cytoplasm, consistent with benign GCT morphology and without atypical features by Fanburg-Smith criteria. **(E)** Hematoxylin and eosin stain (×200): tumor cells circumferentially encasing a peripheral nerve bundle within the lesion in an “onion-skin”-like, concentric distribution external to the perineurium. No tumor-cell penetration of the perineurium and no infiltration of endoneurial axons are identified. This finding represents perineural tumor growth (histogenetic apposition reflecting the Schwann-cell origin of the lesion) rather than perineural invasion as defined for epithelial malignancies (see Section 3.3). The IHC profile of Case 2 (S-100+, CD68+, SOX10+, Pan-CK−, GATA3−, ER/PR/AR−; Ki-67 5–10%) is fully reported in Section 2.2; corresponding IHC images were not retrievable in publication-ready form (see Limitations).

Breast-conserving surgery with intraoperative frozen-section analysis was performed. Frozen section reported “a poorly circumscribed mesenchymal proliferation composed of polygonal cells with eosinophilic granular cytoplasm — favouring a granular cell tumor — but malignancy cannot be confidently excluded on frozen-section morphology alone.” In view of family insistence on definitive axillary management at the index operation, and the inability of frozen section to confidently exclude malignancy, sentinel lymph node biopsy (SLNB) was performed in continuity, with explicit additional informed consent. Final paraffin pathology demonstrated a poorly circumscribed mass measuring 2.5 × 2.0 × 1.8 cm with infiltrative borders. Tumor cells were arranged in nests and cords with abundant eosinophilic granular cytoplasm and small, regular, round-to-oval nuclei (**Figure 2A**). IHC showed diffuse strong cytoplasmic and nuclear positivity for S-100 (**Figure 2B**), diffuse cytoplasmic positivity for CD68 (**Figure 2C**), and additional positivity for SOX10 (image not shown — see Limitations), vimentin, and neuron-specific enolase. The tumor was negative for Pan-CK, EMA, GATA3, TRPS1, GCDFP-15, ER, PR, and AR. The Ki-67 proliferation index was 3–5%. Applying the Fanburg-Smith histological criteria for malignancy (12), the tumor met 0 of 6 criteria and was classified as histologically benign. All surgical margins were tumor-free (closest margin > 5 mm). Both sentinel lymph nodes showed reactive follicular and sinusoidal hyperplasia without metastatic disease.

The patient recovered uneventfully and was discharged on postoperative day 4. Structured surveillance with biannual clinical examination and breast ultrasound was instituted. At 12-month follow-up, she remains disease-free on clinical, sonographic, and mammographic assessment.

### 2.2 Case 2: GCTB with histologically demonstrated peripheral nerve encasement

A 38-year-old premenopausal woman was referred in 2022 for evaluation of a right breast nodule. She had a notable surgical history of two prior excisions for histologically confirmed fibroadenoma at the identical anatomical site (1 o’clock position of the right breast) performed approximately 14 and 8 years previously at outside institutions; she had no personal or family history of breast malignancy. No causal or pathogenetic association between fibroadenoma and granular cell tumour has been established in the literature, and the recurrent nodularity at this site is most parsimoniously attributed to two histologically distinct lesions arising sequentially rather than to a shared aetiology; the prior fibroadenoma history is reported here for completeness of the surgical record. Menarche was at age 12, with one full-term delivery and no hormonal contraception exposure. Clinical examination identified a small, firm, mobile nodule at the prior surgical site, without overlying skin changes or axillary lymphadenopathy.

Targeted breast ultrasound demonstrated an irregular hypoechoic nodule measuring 0.9 × 0.8 × 0.9 cm with posterior acoustic shadowing, classified as BI-RADS 4a. After discussion of the diagnostic and therapeutic options — including conventional open excisional biopsy and minimally invasive ultrasound-guided vacuum-assisted biopsy (VAB) — the patient elected the latter, prioritising minimisation of additional surgical trauma at the previously operated site. Ultrasound-guided VAB was performed using the Mammotome system (Devicor Medical Products) under local anaesthesia. The entire imaging-visible lesion was excised, with real-time sonographic confirmation that no residual hypoechoic tissue was detectable at the conclusion of the procedure.

Histopathological examination demonstrated round to polygonal tumor cells with abundant eosinophilic granular cytoplasm and small, uniform nuclei, arranged in nests and cords within a fibrous stroma (**Figure 2D**) — morphology consistent with benign GCT. IHC confirmed the diagnosis (S-100+, CD68+, SOX10+, Pan-CK−, GATA3−, ER/PR/AR−), with a Ki-67 proliferation index of 5–10%. By Fanburg-Smith criteria the lesion met 0 of 6 criteria and was classified as benign (12).

A morphologically striking and diagnostically informative finding was the demonstration of tumor cells circumferentially encasing peripheral nerve bundles within the lesion (**Figure 2E**). Multiple sections showed concentric, “onion-skin”-like wrapping of small nerve fascicles by sheets of granular tumor cells external to the perineurium, without histological evidence of tumor-cell penetration of the perineurium or invasion of the endoneurium. This intimate apposition of granular cells to peripheral nerves represents a histogenetic relationship characteristic of Schwann-cell-derived neoplasms and is consistent with the established neural origin of GCT (3, 11). It is conceptually and prognostically distinct from perineural invasion (PNI), the latter being a hallmark of epithelial malignancies (13) — a distinction expanded in Section 3.3.

In view of (i) histologically confirmed benign GCTB with 0/6 Fanburg-Smith criteria, (ii) macroscopic and sonographic confirmation that the entire imaging-visible lesion had been excised at VAB, and (iii) absence of clinical, sonographic, or mammographic evidence of residual disease at three months, VAB was regarded as a diagnostic excisional procedure rather than a definitive oncological treatment; because VAB does not yield an inked specimen and cannot provide formal margin assessment, no claim of definitive local control was made. Accordingly, the patient was enrolled, after multidisciplinary discussion and explicit informed consent, in an intensified structured surveillance protocol (biannual clinical examination and ultrasound for two years, followed by annual review with mammography) to detect any residual or recurrent disease at the earliest possible point. At 36-month follow-up, she remains disease-free on clinical, sonographic, and mammographic assessment, without any evidence of residual or recurrent tumor at the VAB cavity.

### 2.3 Timeline

The chronological sequence of key clinical events for both cases is summarized in **Table 1**.

**Table 1:** Timeline of key clinical events for both cases.

Case	Time point	Event
Case 1	October 2024	Presentation with left axillary tail mass; multimodal imaging assessed as BI-RADS 5 with washout-pattern axillary lymph node; MDT recommended CNB, declined by patient (written informed refusal documented).
	December 2024	Breast-conserving surgery + sentinel lymph node biopsy; final pathology: benign GCTB (0/6 Fanburg-Smith criteria) with reactive nodal hyperplasia.
	December 2025	12-month follow-up: no clinical, sonographic, or mammographic recurrence.
Case 2	Approx. 14 years prior	First fibroadenoma excision (right upper inner quadrant, outside institution).
	Approx. 8 years prior	Second fibroadenoma excision at the same site (outside institution).
	November 2022	Ultrasound-guided vacuum-assisted biopsy (Mammotome): benign GCTB with histological encasement of peripheral nerve bundles.
	November 2025	36-month follow-up: no clinical, sonographic, or mammographic recurrence.

CNB, core needle biopsy; GCTB, granular cell tumor of the breast; MDT, multidisciplinary team.

## 3 Discussion

The principal clinicopathological characteristics of the two cases are compared in **Table 2**. The two cases reported here illustrate two clinically consequential dimensions of GCTB that warrant emphasis in contemporary breast practice: the diagnostic trap of axillary tail GCTB radiologically mimicking node-positive invasive carcinoma, and direct histological evidence of perineural tumor growth that supports the Schwann-cell origin of the tumor. Taken together, the unifying message of this report is a single clinical principle: in any suspicious axillary tail mass, a benign Schwann-cell-derived tumour cannot be distinguished from invasive carcinoma on imaging, so preoperative tissue diagnosis — not the choice of any particular biopsy or excision technique — is the decisive step that prevents overtreatment. The two cases, although managed by different operative routes, converge on this same point from opposite directions: Case 1 shows the uncertainty created when tissue diagnosis is deferred, and Case 2 shows how a minimally invasive diagnostic excision, coupled with surveillance, can establish the diagnosis with minimal morbidity.

**Table 2:** Comparison of clinicopathological characteristics.

Characteristic	Case 1	Case 2
Age (years)	53	38
Menopausal status	Postmenopausal	Premenopausal
Tumor location	Left axillary tail (2 o’clock)	Right upper inner quadrant (1 o’clock)
Tumor size (cm)	2.5 × 2.0 × 1.8	0.9 × 0.8 × 0.9
BI-RADS category	5 (mammography)/4c (ultrasound)	4a (ultrasound)
Prior breast surgery	None	Two fibroadenoma excisions (≈14 and ≈8 years prior)
Lymph node status	Radiologically suspicious; pathologically reactive hyperplasia	No abnormality
Diagnostic method	Postoperative pathology (CNB declined by patient)	Vacuum-assisted biopsy (Mammotome)
Surgical procedure	Breast-conserving surgery + SLNB	VAB excision only (diagnostic excisional procedure; not definitive treatment)
Pathological diagnosis	Benign GCTB; margins negative (> 5 mm)	Benign GCTB
Key immunohistochemistry	S-100(+), CD68(+), SOX10(+), Pan-CK(−), GATA3(−), ER/PR/AR(−); Ki-67: 3–5%	S-100(+), CD68(+), SOX10(+), Pan-CK(−), GATA3(−), ER/PR/AR(−); Ki-67: 5–10%
Fanburg-Smith criteria	0 of 6	0 of 6
Follow-up duration	12 months	36 months
Recurrence	None	None

BI-RADS, Breast Imaging Reporting and Data System; CNB, core needle biopsy; GCTB, granular cell tumor of the breast; SLNB, sentinel lymph node biopsy; VAB, vacuum-assisted biopsy.

### 3.1 Axillary tail GCTB: a diagnostic trap on multimodal imaging

Case 1 illustrates the most clinically dangerous presentation of GCTB: an axillary tail lesion simultaneously displaying a BI-RADS 5 spiculated primary mass with skin retraction and a small but radiologically suspicious ipsilateral axillary lymph node. Each feature, in isolation, would prompt strong suspicion of invasive carcinoma; their combination produced a preoperative picture clinically indistinguishable from cT2N1M0 breast carcinoma. Final pathology demonstrated a benign GCTB with reactive nodal hyperplasia.

The radiological mimicry of malignancy by GCTB is well documented across modalities (7, 8). The principal histopathological correlate of these aggressive-appearing imaging features is the infiltrative tumor border and dense fibrous stroma of GCTB (**Figures 2A, D**), which together account for the spiculated mammographic appearance (**Figure 1A**), the marked posterior acoustic shadowing on ultrasound (**Figure 1B**), and the heterogeneous enhancement pattern on MRI (**Figure 1C**). With respect to the kinetic-curve interpretation in Case 1, type II (plateau) and type III (washout) curves are conventionally classified per the ACR BI-RADS MRI lexicon. In the multicentre ACRIN-derived analyses of MRI lesion features, plateau kinetics carry an intermediate-to-high positive predictive value for malignancy (rather than being a definitive malignant marker), while washout kinetics show the strongest individual association with malignancy among kinetic descriptors (14). In Case 1, both elements were present (plateau in the primary lesion, washout in the node), producing a composite picture with a very high pre-test probability of malignancy that nonetheless proved misleading.

In the axillary tail location, the diagnostic difficulty is compounded by two additional factors. First, the proximity to level I axillary lymph nodes means that any reactive lymphadenopathy in the drainage territory is readily interpreted as nodal metastasis. In Case 1, the index lymph node measured only 0.6 × 0.4 cm and showed washout kinetics on the ROI displayed in **Figure 1D**, yet SLNB ultimately demonstrated reactive hyperplasia. In sub-centimetre nodes, partial-volume effects further limit the reliability of kinetic-curve analysis; the kinetic pattern alone should therefore not be regarded as definitive evidence of metastasis. Second, the axillary tail of Spence is a recognised anatomical location both for carcinoma arising directly from the axillary tail (CATS) and for invasive carcinoma arising in ectopic axillary breast tissue (9, 10) — entities that are radiologically indistinguishable from axillary tail GCTB and yet mandate fundamentally different oncological management. Both must be explicitly considered in the differential of any suspicious axillary tail mass.

### 3.2 Preoperative tissue diagnosis: the indispensable diagnostic pivot

The definitive solution to this diagnostic challenge is preoperative CNB with a targeted IHC panel. The characteristic immunohistochemical phenotype of GCTB — diffuse strong positivity for S-100, SOX10, and CD68, with negativity for epithelial markers and hormone receptors — provides a robust separation from primary breast carcinoma and from ectopic axillary breast carcinoma (2, 5, 7). The histological differential is equally important on routine H&E sections. The two invasive breast carcinoma subtypes whose abundant, eosinophilic, granular cytoplasm most closely mimics that of GCT are invasive apocrine carcinoma — in which the granular, PAS-positive “type A” cells predominate — and the apocrine (histiocytoid) variant of invasive lobular carcinoma (2, 3). Both, however, are reliably separated from GCTB by immunohistochemistry: in contrast to the S-100+/SOX10+/CD68+ and Pan-CK−/EMA− phenotype of GCTB, these carcinomas are cytokeratin- and EMA-positive and characteristically express GCDFP-15, androgen receptor, and GATA3, while remaining S-100/SOX10 negative. Because molecular-apocrine carcinomas are frequently ER/PR-negative, the discrimination from GCTB cannot rest on hormone-receptor status alone; the combination of epithelial markers (Pan-CK, GATA3) with apocrine markers (GCDFP-15, AR) is decisive. Notably, the panel performed in Case 1 — which included GCDFP-15, AR, and GATA3, all negative — already excludes these apocrine mimics on the surgical specimen. Operationally, a minimum CNB panel for any suspicious axillary tail lesion should include S-100, SOX10, CD68, Pan-CK, GATA3, and ER/PR. GCTB is expected to be Pan-CK−, GATA3−, ER/PR/AR−, S-100+, SOX10+, and CD68 +. By contrast, invasive carcinoma arising in ectopic axillary breast tissue is expected to be Pan-CK+ and GATA3+ (GATA3 expression in > 90% of primary and metastatic breast carcinomas) (15), with ER/PR positivity in the majority of luminal cases, and consistently S-100/SOX10 negative. This binary IHC contrast renders the differential operationally reliable on a CNB specimen.

In Case 1, the patient declined CNB despite formal MDT recommendation, two documented re-counselling encounters, and a co-signed informed refusal. This case nevertheless illustrates an important practice point: the rationale for preoperative tissue diagnosis must be reinforced across multiple encounters, particularly when imaging suggests advanced disease, because direct surgical management without CNB exposes patients to potentially unnecessary procedures — including axillary lymph node dissection and adjuvant systemic therapy — on the basis of imaging alone. A pragmatic diagnostic pathway for any BI-RADS 4–5 axillary tail lesion should therefore include (i) explicit consideration of GCTB, CATS, and ectopic axillary breast carcinoma in the differential at the initial imaging report; (ii) MDT-driven recommendation for CNB before surgical planning; (iii) the focused IHC panel outlined above; and (iv) re-counselling with explicit documentation if CNB is initially declined.

### 3.3 Perineural tumor growth versus perineural invasion: a critical conceptual distinction

The intimate apposition of granular tumor cells to peripheral nerve bundles in Case 2 (**Figure 2E**) requires careful conceptual interpretation. Perineural invasion (PNI), as defined by Liebig and colleagues and now widely accepted in surgical pathology, denotes either neoplastic infiltration of the epineurium, perineurium, or endoneurium of a nerve by carcinoma cells, or tumor cells closely encircling at least 33% of the nerve circumference; PNI is recognised as a route of metastatic spread in epithelial malignancies — most prominently pancreatic, colorectal, prostatic, and head-and-neck carcinomas — and is associated with poor prognosis and decreased survival (13). PNI in this sense is fundamentally an oncogenic invasion of nerve by a non-neural neoplasm.

The phenomenon documented in our second case is conceptually different. GCT is itself a Schwann-cell-derived peripheral nerve-sheath neoplasm; tumor cells in this disease are derived from the very cellular constituents of the nerve. The morphological picture in **Figure 2E** — concentric, “onion-skin”-like wrapping of small peripheral nerve fascicles by granular tumor cells external to the perineurium, without identifiable penetration of the perineurium or infiltration of endoneurial axons — therefore represents a histogenetic apposition reflecting the neurotropic growth pattern intrinsic to Schwann-cell-derived neoplasms, rather than oncogenic invasion. We deliberately use the term “perineural tumor growth” (or “perineural encasement”) rather than “perineural invasion” to avoid this conceptual confusion and the inappropriate negative prognostic connotation that PNI carries in epithelial cancer. The clinical relevance of this distinction is twofold: first, in a benign Schwann-cell-derived tumor, perineural encasement does not imply an aggressive biological course or worsened prognosis; second, it does not, in isolation, mandate radical surgical clearance beyond what is required to achieve negative margins for the GCT itself.

The histological observation in Case 2 nonetheless contributes meaningful evidence. Direct morphological documentation of tumor cells densely encasing peripheral nerve bundles in GCTB is infrequent in the breast literature and provides individual-case corroboration of the Schwann-cell histogenesis already established at the molecular level by the ATP6AP1/ATP6AP2 work of Pareja and colleagues (4). It also offers a histogenetic explanation for the characteristic infiltrative tumor border observed in GCTB (**Figure 2A**), which underlies the spiculated radiological appearance and the difficulty of achieving clear surgical margins in advanced lesions. This infiltrative growth — even though benign — provides the surgical rationale for the established principle that, for histologically benign GCTB, negative margins remain the standard of care: published series consistently identify positive or close margins as the principal risk factor for local recurrence (6, 16).

### 3.4 Ancillary considerations: Ki-67, frozen section, and minimally invasive management

Three ancillary points merit brief discussion. First, Ki-67 was 3–5% in Case 1 and 5–10% in Case 2. Although both values are comfortably low and the Fanburg-Smith classification — which does not formally incorporate a Ki-67 threshold — was met by 0 of 6 criteria in each case (12), Ki-67 nonetheless retains supportive (though not definitive) value in the benign-versus-malignant assessment of GCT. Nasser and colleagues showed that Ki-67 expression is significantly higher in atypical and malignant GCTs than in benign tumors and proposed a simplified two-tier system based on necrosis and mitotic activity, retaining a complementary role for Ki-67 in difficult cases (17). The low Ki-67 indices in both of our cases are therefore reassuring but should not be regarded as a stand-alone marker of benignity.

Second, the role of intraoperative frozen section in suspected GCTB is intrinsically limited. As exemplified by Case 1, frozen-section morphology can readily suggest a granular cell tumor but cannot reliably exclude malignancy in the absence of immunohistochemistry, which is not feasible on frozen sections. This limitation should be communicated explicitly during preoperative counselling so that the clinical decision about concomitant axillary surgery is not made on overly optimistic expectations of intraoperative pathology.

Third, the role of minimally invasive techniques such as ultrasound-guided VAB in the management of small, histologically confirmed benign GCTB merits cautious consideration. Vacuum-assisted excision of benign breast mass lesions is associated with high complete-resection rates and low recurrence rates in meta-analytic data across diverse benign histologies (18). In Case 2, the combination of (i) histologically confirmed benign GCTB on the VAB specimen itself, (ii) sonographic confirmation that no residual hypoechoic tissue remained at the procedure end, and (iii) sustained absence of clinical or imaging recurrence over 36 months illustrates — at the level of an individual case — only that VAB can serve as an adequate diagnostic excisional procedure in carefully selected sub-centimetre benign GCTB. We wish to state explicitly that VAB should not be regarded as a definitive therapeutic modality for GCTB: because it does not yield an inked-margin specimen, it cannot establish complete oncological clearance, and the margin-negative excision that constitutes the standard of care for GCTB cannot be confirmed by VAB. In our case, the favourable outcome therefore reflects intensified imaging surveillance rather than any proven curative property of the technique. Accordingly, where VAB is used it should be confined to small, IHC-confirmed benign lesions in patients enrolled in an intensified imaging surveillance protocol, and should be understood as a diagnostic — not a curative — intervention; broader prospective evaluation is required before any therapeutic role for VAB in GCTB could be considered, and this falls outside the principal scope of the present report.

### 3.5 Limitations

Several limitations of the present report should be acknowledged. First, this is a two-case report from a single institutional setting and its findings are intended to be illustrative rather than statistically representative of the wider GCTB population. Second, in Case 1, the patient’s decision to decline CNB precluded direct comparison of preoperative biopsy and final surgical pathology; the diagnostic value of CNB is therefore inferred from the literature rather than directly demonstrated in this case. Third, although both cases were positive for SOX10 in the institutional IHC panel — SOX10 currently being a key Schwann-cell-lineage marker and an important discriminator from epithelial neoplasms — representative SOX10 immunostain images were not retrievable from the digital pathology archive in publication-ready form at the time of manuscript preparation; only the S-100 and CD68 panels (**Figures 2B, C**) are therefore illustrated for Case 1. For the same archival reasons, Case 2 is illustrated only by H&E images (**Figures 2D, E**); however, both cases satisfied a fully reported IHC panel in the pathology record (Section 2.1 and 2.2). Fourth, the documentation of perineural tumor growth in Case 2 is morphological rather than ultrastructural or molecular: although the granular-cell phenotype and the surrounding stromal context are unambiguous on H&E, specific nerve-element immunostains (e.g. neurofilament or EMA) were not performed on the corresponding sections to formally label the encircled structures as peripheral nerve fascicles. Electron microscopy and ATP6AP1/ATP6AP2 mutational analysis were likewise not undertaken. Fifth, the follow-up periods (12 and 36 months) are relatively short for definitive assessment of long-term oncological outcomes, and continued surveillance is warranted in both patients.

## 4 Conclusion

Granular cell tumor of the breast in the axillary tail can present with a composite multimodal imaging picture clinically indistinguishable from a locally advanced node-positive invasive breast carcinoma and is a clinically important diagnostic mimic in this anatomical setting. Preoperative core needle biopsy with a targeted immunohistochemistry panel — explicitly designed to discriminate GCTB from carcinoma of the axillary tail of Spence and from ectopic axillary breast carcinoma — should be strongly recommended to avoid overtreatment and vigorously advocated even when imaging features strongly suggest malignancy. Although CNB was declined in Case 1, that case demonstrates precisely the diagnostic uncertainty and risk of overtreatment that arise when tissue diagnosis is omitted, and thereby reinforces — rather than weakens — the argument for obtaining it; the VAB performed in Case 2 should be understood as a diagnostic excisional procedure under intensified surveillance, not as a definitive therapeutic alternative. Direct histological demonstration of tumor-cell encasement of peripheral nerve bundles, as documented in our second case, provides individual-case morphological support for the established Schwann-cell origin of the tumor; this finding should be conceptually distinguished from perineural invasion in epithelial malignancies and reinforces the rationale for adequate (rather than radical) surgical margins, in light of the intrinsic neurotropic growth pattern of this benign entity.

## Data Availability

The original contributions presented in the study are included in the article/supplementary material. Further inquiries can be directed to the corresponding authors.
